# Larval Geoduck (*Panopea generosa*) Proteomic Response to Ciliates

**DOI:** 10.1038/s41598-020-63218-x

**Published:** 2020-04-08

**Authors:** Emma Timmins-Schiffman, José M. Guzmán, Rhonda Elliott Thompson, Brent Vadopalas, Benoit Eudeline, Steven B. Roberts

**Affiliations:** 1 University of Washington, Department of Genome Sciences, 3720 15th Ave NE, Seattle, WA 98195 United States; 2 University of Washington, School of Aquatic and Fishery Sciences, 1122 Boat St., Seattle, WA 98195 United States; 3 Taylor Shellfish Hatchery, 701 Broadspit Rd., Quilcene, WA 98376 United States; 4 Mason County Public Health, 415N 6th St., Shelton, WA 98584 United States

**Keywords:** Climate-change ecology, Proteomics, Animal physiology

## Abstract

The innate immune response is active in invertebrate larvae from early development. Induction of immune response pathways may occur as part of the natural progression of larval development, but an up-regulation of pathways can also occur in response to a pathogen. Here, we took advantage of a protozoan ciliate infestation of a larval geoduck clam culture in a commercial hatchery to investigate the molecular underpinnings of the innate immune response of the larvae to the pathogen. Larval proteomes were analyzed on days 4–10 post-fertilization; ciliates were present on days 8 and 10 post-fertilization. Through comparisons with larval cultures that did not encounter ciliates, proteins implicated in the response to ciliate presence were identified using mass spectrometry-based proteomics. Ciliate response proteins included many associated with ribosomal synthesis and protein translation, suggesting the importance of protein synthesis during the larval immune response. There was also an increased abundance of proteins typically associated with the stress and immune responses during ciliate exposure, such as heat shock proteins, glutathione metabolism, and the reactive oxygen species response. These findings provide a basic understanding of the bivalve molecular response to a mortality-inducing ciliate and improved characterization of the ontogenetic development of the innate immune response.

## Introduction

Bivalve invertebrates possess an innate immune response that can be triggered by biotic threats, including pathogens. There are three steps in the innate immune response including immune recognition, immune signaling, and immune effectors^1^. Once the response is activated, proteins are upregulated that are essential in immune defense and pathogen removal. Adult bivalves are able to activate the entire suite of proteins necessary for a successful immune response, but larvae may have less developed response that makes them more vulnerable to infection and mortality^2^. Increased vulnerability has been observed in differential mortality rates between developmental stages of the same bivalve species upon exposure to the same pathogen^3,4^.

The larval bivalve immune system may begin developing in the embryo within hours post-fertilization^2^. Gene transcripts and proteins involved in the larval immune response are expressed throughout developmental stages, and likely play important roles in larval development as dramatic tissue restructuring occurs^2,5–10^.

Ciliates are widespread in aquatic systems and are known harmless parasites of many species of bivalves, but some species are capable of causing significant mortality. Ciliate parasites have been observed in multiple species of bivalves in hatchery and in wild settings^11–13^. In zebra mussels, increased ciliate infestations were associated with moribund/dead mussels, but the authors hypothesized that the ciliates opportunistically feed on dead and dying tissue from a distinct, primary infection^11^. Ciliate outbreaks in hatcheries can also occur when bacterial communities are unchecked by antibiotics^7^. Bacterivorous ciliates can impact the settlement rates of invertebrate larvae through secondary impacts on water currents above the settlement surface or selective grazing of specific bacteria^14^. Ciliates are known to be more prevalent in hatcheries^12^ where they enter in bulk water and persist in the system^15^.

The geoduck clam (*Panopea generosa*) is a burrowing hiatellid clam found in low intertidal and subtidal sediments throughout the Northeast Pacific coast, including the US (Alaska, Washington, California), Canada (British Columbia), and Mexico (North Baja Pacific Coast)^16–18^. In addition to their economic importance, geoduck are abundant and essential environmental engineers in their natural habitat^19,20^. Geoduck larvae and adults are susceptible to parasites in the Puget Sound, WA region^21,22^. Having established a baseline proteome for geoduck larval development in previous work^23^, we sought to better characterize the geoduck immune response to ciliate pathogens.

In this study, we take advantage of an opportunistic infestation of a putative parasitic ciliate in a commercial hatchery to characterize for the first time the immune response of geoduck clam larvae during veliger development (days 4–10 post-fertilization). This was accomplished using liquid chromatography coupled with high-resolution tandem mass spectrometry that allowed us to build a proteomic profile of larval development and characterize the induced innate immune response in the veliger larvae.

## Results

### Larval growth and mortality

By day 4 post-fertilization, geoduck larvae had an average shell size of 131 µm. This increased to 146 µm on day 6 and 161 µm on day 8 post-fertilization. At day 4 post-fertilization, 3.1 million larvae were stocked in each tank. By day 10 post-fertilization there were 516,000 larvae in one tank and 1.3 million in the other. Ciliates were observed in the larval samples by day 8 post-fertilization; by day 10, there were high numbers of ciliates in the larval culture accompanied by high larval mortality.

### Proteomics of larval development

**Figure 1:**
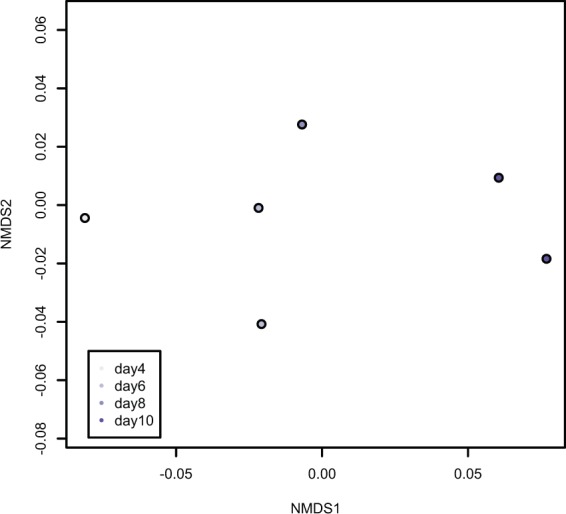
Nonmetric multidimensional scaling plot of larval proteomes (N = 6,133 proteins) across days 4 through 10 post-fertilization. The points are lightest on day 4 and darkest on day 10. The two points representing proteomes on day 8 are directly on top of each other.

The abundance of proteins in the geoduck larval proteomes (N = 6,133 proteins; Supplementary Table S1) are significantly distinct from each other across days 4 through 10 post-fertilization (ANOSIM R = 0.8889, p-value = 0.005; Fig. 1). Four proteins are strongly, positively weighted on NMDS1 axis and thus contribute significantly to proteomic changes over time - elongation factor 1 a (geoduck protein ID in Supplementary Table S1 DN61152_c1_g2_i1::g.31740), deleted in malignant brain tumors 1 protein (DN55033_c0_g1_i1::g.9300), peptidyl-prolyl cis-trans isomerase (DN40375_c0_g1_i1::g.39279), and tubulin alpha chain (DN61033_c0_g2_i3::g.6327).

The strongest signal in the differentially abundant enrichment analysis comes from the changing abundance of proteins involved in translation over time. Many of these proteins increase in abundance over time. Between days 6 and 8 post-fertilization, proteins at elevated abundance on day 8 (n = 38 proteins) contribute to the enrichment of GO terms translational elongation (biological process, BP), and translational elongation factor activity (molecular function, MF). Between days 8 and 10, proteins at relatively higher abundance on day 10 (n = 138 proteins) are responsible for the enriched terms translation (BP), translational elongation (BP), ribosome (cellular component, CC), cytosolic large ribosomal subunit (CC), small ribosomal subunit (CC), structural constituent of ribosome (MF), and rRNA binding (MF).

### Proteomic profile of larval development

Proteins regulating the progress of larval development were identified through comparison with similar proteomes from larvae raised at lower pH that did not experience ciliate-induced mortality. Functional categories identified in both datasets were determined to be involved in development, as opposed to the stress response. Diverse protein groups made up the full suite of differentially abundant proteins across developmental time. Proteins at elevated abundance on day 6 compared to day 8 (n = 28 proteins) included those involved in muscle activity, cytoskeletal structure, amino acid degradation, and carbohydrate metabolism. Proteins involved in muscle activity were four isoforms of twitchin (DN4488_c0_g1_i1::g.37991, DN84882_c0_g1_i1::g.7474, DN37585_c0_g1_i1::g.30792, DN54513_c0_g1_i1::g.20073). Cytoskeletal structure proteins included an axonemal 84 kDa protein (DN52327_c0_g1_i1::g.16221), two isoforms of spectrin beta chain (DN49196_c0_g1_i1::g.12832, DN63615_c0_g1_i4::g.19798), and dystonin (DN64009_c1_g1_i4::g.9078). An enzyme involved in glycine degradation (glycine cleavage system P protein - DN58628_c5_g1_i2::g.33280) and lysine degradation (alpha-aminoadipic semialdehyde synthase - DN102693_c0_g1_i1::g.40254) were elevated on day 6. The up-regulation of carbohydrate metabolism was supported by the inclusion of malic enzyme (DN55577_c7_g1_i1::g.30103), endoglucanase (DN59810_c3_g1_i1::g.27515), and transketolase (DN62420_c0_g1_i1::g.26365).

On day 8, proteins at increased abundance were involved in processes such as embryonic development, translation, stress response, and the cell cycle. Mesenchyme-specific cell surface glycoprotein (compared to day 6, DN60034_c1_g1_i1::g.37906), agrin (compared to days 6 and 10, DN50844_c0_g1_i1::g.3048), and delta-like protein (compared to day 10, DN64019_c1_g1_i2::g.9096) were at elevated abundance on day 8 and are responsible for tissue development and neuromuscular junctions during embryogenesis. Several proteins involved in protein translation, transport, and degradation were at higher levels on day 8, including ribosomal proteins (e.g., DN55459_c0_g2_i2::g.4318), elongation factors (e.g., DN61142_c1_g2_i1::g.31740), nucleolar protein 56 (DN57041_c0_g2_i1::g.3336), RNA polymerase-II associated factor (DN57191_c0_g1_i2::g.28950), and cathepsins (e.g., DN54782_c0_g1_i1::g.5290). On day 8 there may have been an up-regulation of the cell cycle with evidence from increased abundance (compared to day 10) of proteins involved in cell cycle control and the cytoskeleton (e.g., centrosomal protein of 78 kDa - DN44281_c0_g1_i2::g.1342, nuclear protein MDM1 - DN64001_c0_g1_i1::g.14401, antigen KI-67 - DN63195_c0_g2_i2::g.25524, suppressor of G2 allele of SKP1-like protein - DN63050_c5_g2_i1::g.279). Apoptosis proteins were at elevated abundance, including cell division cycle and apoptosis regulator protein 1 (compared to day 10, DN55421_C0_g1_i1::g.4308) and programmed cell death protein 4 (compared to days 6 and 10, DN60246_c2_g3_i2::g.7173). Cilia biogenesis was also important at this time point with an increased abundance of proteins such as centrin-4 (DN30560_c0_g1_i2::g.39913) and dynein heavy chain 7, axonemal (DN52275_c0_g1_i1::g.16099), cilia- and flagella-associated protein 58 (DN63766_c0_g1_i4::g.4722), and intraflagellar transport protein 57-like (DN62855_c0_g2_i1::g.20768). Four of the proteins that were at elevated abundance on day 8 were glycoproteins: mesenchyme-specific cell surface glycoprotein (DN60034_c1_g1_i1::g.37906), prosaposin (DN63880_c2_g1_i3::g.30762), agrin (DN50844_c0_g1_i1::g.3048), and fibronectin type-III domain-containing protein (DN55720_c0_g1_i1::g.14093).

The most significant proteomic signal on day 10 was a surge in abundance of ribosomal proteins with other proteins at higher abundance in categories such as protein folding, tissue growth/restructuring, and energy metabolism. Dozens of ribosomal proteins, several elongation factors, and other proteins associated with translation were at increased abundance on day 10. As a likely balance to the surge of new proteins, several proteins involved in protein degradation (aminopeptidase N - DN60972_c0_g2_i1::g.12028, cysteine peptidase - DN24514_c0_g1_i1::g.3095) and protein folding (a suite of heat shock proteins - e.g., DN56353_c0_g1_i1::g.29541, peptidyl-prolyl cis-trans isomerase - DN40375_c0_g1_i1::g.39279, calreticulin - DN42620_c0_g1_i1::g.2821, and glucose-regulated protein 78 - DN41738_c0_g1_i2::g.3144) were also detected at elevated levels on day 10. There is also evidence of tissue restructuring with an increase in abundance of proteins involved in muscle growth (kyphoscoliosis peptidase - DN54342_c0_g1_i1::g.35621, actin - DN40952_c0_g1_i1::g.3693, twitchin - DN84882_c0_g1_i1::g.7474), vascularization (basement membrane-specific heparan sulfate proteoglycan core protein - DN1574_c0_g1_i1::g.30025), and embryogenesis (multiple epidermal growth factor-like domains protein - DN61224_c0_g1_i1::g31991). There was also an increase in abundance of proteins involved in metabolism and energy production: acetyl-CoA C-acyltransferase, succinate-CoA ligase [ADP/GDP-forming] subunit alpha, ATP/ADP translocase, and adenosine kinase (DN65641_c0_g1_i1::g.38953, DN5111_c0_g1_i1::g.3560, DN31265_c0_g1_i1::g.39364, DN86572_c0_g1_i1::g.31553).

### Proteomic profile of larval immune response

**Figure 2:**
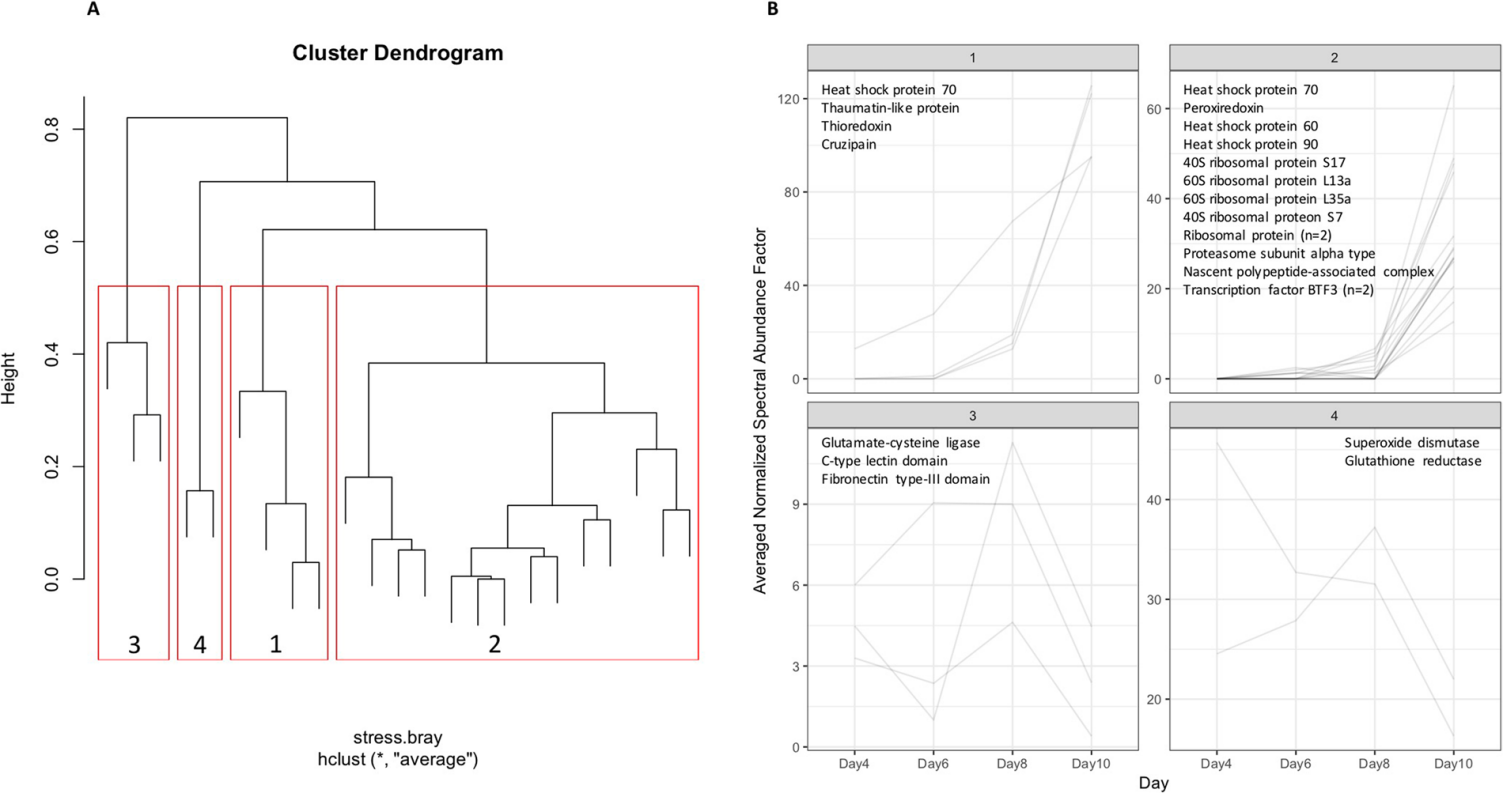
Proteins hypothesized to be involved in the larval stress response to ciliate infestation. The clustered protein group dendrogram (panel A) shows the 4 clusters of proteins in red boxes with the cluster number at the bottom of each box. Panel B shows the abundance profile for each protein across day 4–10 post-fertilization with abundance plots grouped by cluster (cluster number is in the header of each plot). Annotations for the proteins in each plot are listed within each plot. All proteins are likely geoduck larval proteins except for cruzipain (cluster 1), which is a parasitic virulence protein.

There were 855 proteins identified in the pH 8.2 dataset that were not detected in larvae reared in parallel at lower pH where there was no ciliate presence. Some of these proteins would have been detected in the current dataset because of their role in ciliate response; others may have been unique to this dataset simply due to the difference in ambient pH of the larval culture. In the enrichment analysis, compared to all proteins in the pH 8.2 dataset, enriched terms included integral component of membrane (CC), membrane (CC), regulation of transcription DNA-templated (BP), and DNA-binding transcription factor activity (MF). Eleven of these proteins were significantly differentially abundant (Fig. 2), 10 of which were highest at day 10 and most of which are ribosomal proteins.

On day 8, ciliates were first observed in the larval culture. In addition to the proteins described in the previous section, stress response proteins also increased on day 8 compared to days 6 and 10, including two isoforms of heat shock protein 70 (DN56353_c0_g1_i1::g.29541, DN56353_c0_g1_i2::g.29542), a thaumatin-like protein (DN55757_c0_g1_i1::g.14062), glutamate-cysteine ligase catalytic subunit (DN62819_c0_g1_i1::g.20739), glutathione peroxidase (DN60231_c1_g1_i1::g.7155), glutathione reductase (DN61098_c8_g3_i1::g.6407), and superoxide dismutase (DN61210_c0_g1_i3::g.32050) (Fig. 2).

The ciliate infestation had increased by day 10, along with larval mortality (see above). The parasite virulence factor cruzipain (DN76433_c0_g1_i1::g.22190) was first detected on day 8 in the proteomics data and was at significantly elevated abundance by day 10 (Fig. 2).

## Discussion

Geoduck larvae have distinct proteomic profiles based on developmental stage with evidence of a molecular response to protozoan ciliate infestation. Most of the differentially abundant proteins between developmental time points were likely involved in the progression of larval development since they represented similar functional groups to those proteins detected in parallel larval cultures. Eleven larval proteins and one protozoan protein emerged as likely markers of ciliate infestation of larval cultures. Over 800 proteins were detected in the current dataset of larvae reared at pH 8.2 and were not detected at lower pH, which included many proteins involved in protein translation; those that were significantly differentially abundant increased in abundance during the ciliate infestation (days 8 through 10 post-fertilization). This dataset further establishes the timeline of larval molecular development and also elucidates some of the mechanisms deployed by the larval immune response.

Protein abundance patterns across time points in larvae reared at pH 8.2 generally followed the established timeline for geoduck at pH 7.5. Even though we compared larvae across different pH, the larvae reared at pH 7.5 are an appropriate direct comparison for this study because 1) baseline proteomic trends of development were maintained across the two pH, 2) they are from the same spawning event, and 3) the larvae at both pH developed normally (there was no sign of adverse impacts of pH on morphology at pH 7.5). Changes in abundance of proteins involved in protein metabolism and translation dominated the results of proteomic differences across larval development. This trend was apparent in the analysis of the NMDS (Fig. 1), where two of the proteins strongly weighted along axis 1were associated with translation (elongation factor 1a) and protein folding (peptidyl-prolyl cis-trans isomerase). Additionally, many of the differentially abundant proteins between time points were associated with translation and protein metabolism, a trend also observed in geoduck larvae reared at lower pH^23^. The maintenance and upregulation of translation, even under the stress of ciliate infestation, underlines the physiological drive of the developmental processes that rely on new proteins to grow and create new tissue structures, but likely also underpins the immune/stress response to ciliates (see below). At day 6 post-fertilization there was increased abundance of proteins involved in muscle activity, cytoskeletal structure, amino acid degradation, and carbohydrate metabolism. At day 8 post-fertilization, proteins involved in embryonic development, translation, and the cell cycle increased. Increased functional categories at day 10 post-fertilization included translation, along with increased abundances of proteins involved in tissue growth and energy metabolism. A full discussion of the significance of these changes can be found in [23].

Ciliates were detected in the larval culture at day 8 post-fertilization and starting at this time point there was molecular evidence of a larval stress and immune response. Some of these stress response proteins peaked in abundance at day 8 and some at day 10 (Fig. 2), at which point the ciliate infestation was more intense and significant larval mortality was observed. The hypothesized larval proteomic response to ciliates included an increase in abundance of the molecular chaperones heat shock proteins and of proteins involved in the reactive oxygen species (ROS) response. Immune response proteins are known to be active during larval development and metamorphosis^5,24^. Several proteins annotated with GO terms “immune response” and “innate immune response” were detected at pH 8.2 and in the pH 7.5 dataset: interleukin enhancer-binding factor-2 like (DN57893_c2_g2_i1::g.33736), tubulointerstitial nephritis antigen-like (DN61593_c0_g3_i1::g.26870), RPE-spondin (DN63587_c1_g2_i1::g.19583), peptidoglycan-recognition protein (DN54849_c0_g1_i1::g.30972), and protein toll (DN101391_c0_g1_i1::g.40331). The primary role of immune response proteins in development is hypothesized to be recognizing and responding to dying tissue during tissue resorption and organogenesis, but many of these proteins are also induced upon pathogen exposure^2,5–9^. Embryonic immunity is derived from maternal peptides and proteins^2,10^, but, starting at the gastrula and trochophore stages, bivalve larvae produce their own immune response proteins with immune competence reached between the D-hinge and veliger stages^2^.

Heat shock proteins (HSPs) are significant participants in the invertebrate immune response and increased levels of these proteins were detected on days 8 and 10 post-fertilization in this study, with highest abundances at day 10. The suite of differentially abundant heat shock proteins included two isoforms of HSP70, chaperoning HSP60, and HSP90. In marine fish and invertebrates, HSP upregulation occurs in response to any external stressor, including disease^25^, likely as a response to stress-induced tissue and protein damage. These molecular chaperones may play roles in intercellular signaling as well as chaperoning peptides released from dead and dying tissues^25^. This conserved response is activated across bivalve larvae. In veliger Pacific oysters gene expression of *hsp70* increased upon infection by the bacteria *Vibrio corallilyticus*^9^. In oyster larvae untreated with antibiotics, gene expression levels of *hsp70* were elevated a week before a mass mortality event, but decreased immediately before larval mortality occurred^8^. In the current study, geoduck larvae were not fighting off a known bacterial infection, but were responding to attack from ciliates. It may be that an upregulation of heat shock proteins was part of a general immune response upregulation, or a response to the tissue damage incurred by ciliates.

Reactive oxygen species (ROS) are a well-established component of the invertebrate immune response and response to these damaging molecules can be seen in the proteomic profiles of the larvae. Thioredoxin, peroxiredoxin-1, and superoxide dismutase were all detected at elevated abundances on day 8 and/or 10 post-fertilization. These proteins are well-known for their roles in responding to ROS to reduce tissue damage. Additionally, glutamate-cysteine ligase, glutathione reductase and glutathione peroxidase, all part of the glutathione metabolism pathway, were increased on day 8 post-fertilization, compared to day 10. Glutathione is an essential part of the cellular response to ROS to prevent cellular damage^26^. Adult bivalves are known to up-regulate ROS production as a defense mechanism against pathogens^27^ and there is evidence that bivalve larvae do the same as early as the trochophore stage^6,7^. When molluscan hemocytes detect a pathogen, they undergo respiratory burst, which generates free radicals such as ROS^28^. In oyster larvae, gene expression and enzymatic activity of superoxide dismutase, catalase (another ROS defense protein), peroxiredoxin, and the glutathione pathway were all elevated in response to pathogen exposure in veligers^7–9^. Geoduck larvae seem to effect the same molecular cascade of hemocyte respiratory burst, followed by ROS mitigation, in their response to ciliates.

An apparent upregulation of protein translation during the ciliate infestation was evidenced by the differential abundance of several of these proteins during exposure to ciliates and detected only in larvae reared at pH 8.2. Protein translation underlies any type of proteomic upregulation as new proteins are synthesized in response to a stimulus. Protein translation in the eukaryotic host is an essential part of the immune and stress responses^29^. It may be that these differentially abundant proteins (6 ribosomal proteins, proteasome subunit alpha type, nascent polypeptide-associated complex, and 2 transcription factors BTF3) were detected at increased abundance coincident with ciliate presence because they are the foundation of immune physiology upregulation as larvae attempt to defend themselves against the parasite. They may also be evidence of the unfolded protein response and endoplasmic reticulum stress^30^, although many of the molecular hallmarks of this response were not detected in the differentially abundant proteins. It is clear that the larval immune response encompasses a network of proteins that work together to launch a physiological change and that this network extends beyond the typical biomarkers selected for single protein or enzyme assays.

The proteomic signals of a physiological stress response and coincident mass mortality in the geoduck larvae was very likely a direct cause of the ciliate infestation. The evidence for this causal link is the observation of numerous ciliates coincident with the stress response and morality; the known link between ciliates and larval mortality in Pacific Northwest hatcheries^31^; and the increased abundance of the protozoan protein homologous to cruzipain at days 8 and 10 post-fertilization. In larvae reared in the same hatchery over the same period, but at lower pH (7.5 and 7.1), ciliates were not as prevalent as they were at pH 8.2 (unpublished data) and a mass mortality was not observed. Since the larvae across all pH were reared in the same water and a mass mortality was only observed at pH 8.2, it is unlikely that a bacterial or viral pathogen was the cause of larval mortality. A ciliate in the Family Orchitophyridae is a known primary pathogen of bivalve seed and larvae, infection by which leads to significant mortalities^31^. These ciliates were observed to enter oyster seed through the mantle cavity, where they proceeded to feed upon oyster tissue and fluids, destroying organs and leading to death^31^. Coincident with the ciliate infestation, the abundance of the protozoan protease homologous to cruzipain, used in the break-down of host tissues, was detected. Cruzipain is a protein that mediates infection in the parasites of the genus *Trypanosoma*^32^, but a homologous protein has been detected in parasites of divergent taxa as well^33^. A homologue of cruzipain is apparently also present in the ciliates observed in the hatchery to assist in larval tissue breakdown.

In a characterization of larval geoduck proteomes from day 4 through 10 post-fertilization, we were able to detect trends of normal physiological development as well as aberrations caused by a stress response to a ciliate infestation of the culture. Normal development was characterized by proteomic markers of tissue growth and upregulation of metabolism. Once the ciliates were detected, molecular signals of a stress response were observed, especially in the increased abundance of specific protein translation proteins, heat shock proteins and response to oxidative damage. These signals confirm that geoduck larvae have an inducible immune/stress response in the veliger stage, as has been observed in other bivalve larvae^9^. These universal markers can be used to detect and track the health of larval cultures in hatchery settings.

## Methods

### Larval rearing and ciliate outbreak

Broodstock maturation, spawning, and larval development (days 1–10 post-fertilization) occurred in a commercial hatchery in Hood Canal in Washington (USA) per normal operation conditions. Water temperature was maintained at approximately 14 °C, and larvae were reared in two 200 L conical tanks at a density of 70,000 fertilized eggs/L.

Larvae were maintained in seawater treated with Na_2_CO_3_ to maintain a pH of 8.2 and were sampled on days 4, 6, 8, and 10 post-fertilization. However, on days 8 and 10 post-fertilization, a ciliate infestation was observed in both tanks. The infestation was characterized by an increase in number of ciliates observed between days 8 and 10 post-fertilization and increasing levels of larval mortality. After day 10 post-fertilization, the experiment had to be discontinued due to mortality.

Given that ciliate infestation events have become more severe and frequent in bivalve commercial hatcheries in the US Pacific Northwest during recent years, and the cost associated with these outbreaks, we decided to take advantage of this event to characterize the immune/stress response of geoduck clam larvae to this opportunistic parasite ciliate infestation.

### Larval sampling

On days 4, 6, 8 and 10 post-fertilization, approximately 10,000 larvae were collected using a 20 µm mesh screen, rinsed with 70% isopropyl alcohol, and stored at −80 °C for proteomics. Day 4 represented a bulk sample of larvae before they were divided between two tanks; for subsequent time points, larvae were sampled from n = 2 tanks. For each sampling, larval size classes were determined using a series of mesh screens (from 90–200 µm in 20-µm intervals). The number of larvae retained in each screen was estimated by weight based on an established conversion developed in the hatchery (Supplementary Table S2).

Larvae were sampled on days 4, 6, 8, and 10 post-fertilization for proteomic analysis as part of a parallel study at pH 7.1 and 7.5^23^. Ciliate-associated mortality was not observed at either of these lower pH giving us a non-ciliate impacted proteome against which we could compare the proteome of larvae responding to ciliates.

### Protein extraction and LC-MS/MS

Larval proteins were digested and peptides were desalted following^34^. Larval peptides were analyzed on an Orbitrap Fusion Lumos mass spectrometer (Thermo Scientific, Waltham, MA, USA) with a 4.5 cm, 100 µm pre-column and a 26 cm, 75 µm analytical column, both packed in-house with 3 µm C18 Dr. Maisch (Germany) beads. The analytical column was housed in a 50 °C column heater for the duration of the analysis to improve chromatography. Over a 120 minute method, a 90-minute acetonitrile gradient went from 5–30%. In MS1 analysis in the Orbitrap, the resolution was 120 K, scan range was 375–1575 *m/z*, max injection time was 50 ms, and AGC target was 700000. During the MS2 analysis in the IonTrap maximum injection time was 100 ms, AGC target was 2000, and centroid data was collected. The mass spectrometry proteomics data have been deposited to the ProteomeXchange Consortium via the PRIDE^35^ partner repository with the dataset identifier PXD013667.

### Proteomics analysis

All mass spectrometry raw files were searched against a deduced geoduck larvae proteome^36^ with the addition of common laboratory contaminants from bovine, human, and other (cRAPome). Redundancy of sequences in the larval proteome was reduced using CD-hit^37^. Details on reference proteome development are described elsewhere^38^. Comet^39,40^ 2016.01 rev. 3 parameters included concatenated decoy search, mass tolerance of 20 ppm, 2 allowed missed trypsin cleavages, fragment bin tolerance of 1.0005 and offset of 0.4. Peptide and Protein Prophet^41,42^ were run consecutively using xinteract with no probability cut-off to allow for FDR cut-off later in the pipeline. Resulting pep.xml files were analyzed in Abacus^43^ with a FDR cut-off of 0.01 (probability from combined prot.xml file of 0.9) (Supplementary Methods).

Nonmetric multidimensional scaling (NMDS) analysis was performed on all proteins that were inferred across technical mass spectrometry replicates with at least 2 unique peptide spectral matches. Protein abundance data (normalized spectral abundance factor) were log(x + 1) transformed and a Bray-Curtis dissimilarity matrix was calculated in R (https://www.r-project.org/). Eigenvectors were generated using envfit in the vegan package^44^. An eigenvector was considered significant if its axis loading value was >0.9 and its p-value was <0.001. ANOSIM was performed on normalized spectral abundance factor (NSAF) data standardized by row to assess significance of trends observed on the NMDS based on day.

Differentially abundant proteins between consecutive time points (e.g., day 6 vs day 8) were determined using Qspec^45^. This analysis was not done for day 4 since there was just a single data point for that day. For each QSpec analysis, only proteins that had a non-zero sum of spectral counts across replicates were included and spectral counts were summed across technical replicates. Proteins were considered differentially abundant if they had a QSpec log-fold change of at least |0.5| and a z-statistic of at least |2 | .

Based on homology of protein annotations, 11 differentially abundant proteins were considered to be involved in larval response to ciliates and one protein (cruzipain) was likely an indicator of ciliate proliferation. These proteins were grouped using a hierarchical clustering method based on NSAF values over time. NSAF values were averaged across conical replicates and mass spectrometry technical replicates within days and a Bray-Curtis dissimilarity matrix was calculated in R using the average clustering method. Cluster division was based on dendrogram topology (cut-off = 0.5). Cluster abundance traces were visualized using ggplot2^46^.

Enrichment analysis was performed using an in-house Gene Ontology (GO) term enrichment tool described in^47^ via the project-specific portal https://meta.yeastrc.org/compgo_emma_pgenph8/pages/goAnalysisForm.jsp. The over-representation of GO categories (cellular component, biological process, and molecular function) was assessed in proteins that were differentially abundant between days or detected only on a particular day with a p-value cut-off of 1E-1.

Proteins identified in the pH 8.2 dataset were compared with proteins inferred from the low pH study^23^ to identify proteins that may be involved in the immune response to ciliates since no ciliate-induced mortality was observed at lower pH. These proteins were subjected to enrichment analysis and cross-referenced with the differentially abundant proteins.

## Supplementary information

Supplementary information.

Supplementary information 2.

## Data Availability

The proteomic dataset generated during and/or analyzed during the current study is available in the ProteomeXchange Consortium repository via the PRIDE partner repository with the dataset identifier PXD013667.
