# Supplementary material for: Larval Geoduck (Panopea generosa) Proteomic Response to Ciliates
Source: Sci Rep. 2020 Apr 8;10:6042. doi: 10.1038/s41598-020-63218-x (PMC7142153; doi:10.1038/s41598-020-63218-x)
Supplement: Supplementary file 2 — Supplementary information 2. [file 41598_2020_63218_MOESM2_ESM.pdf]

```
#
# ABACUS parameter file
# Generated on: 2016Jun30_1144
#

# Name to give the database
dbName=ABACUSDB

# Name of protXML file corresponding to merged/combined results
combinedFile=/net/gs/vol4/shared/nunnlab/search/emmats/TPP/
2017_July_24_geolarv/cdhit0.9/interact-COMBINED.prot.xml

# The directory that contains the pepXML and protXML files
srcDir=/net/gs/vol4/shared/nunnlab/search/emmats/TPP/
2017_July_24_geolarv/cdhit0.9

# The name of the file where results will be saved to
outputFile=/net/gs/vol4/shared/nunnlab/search/emmats/TPP/
2017_July_24_geolarv/cdhit0.9/ABACUS_cdhit0.9_output.tsv

# The path the the FASTA formatted file used for the original protein
search
# Relative paths are allowed
fasta=/net/gs/vol4/shared/nunnlab/search/emmats/databases/
Pgen_larvae_cd0.9.contam

# The minimum PeptideProphet score the best peptide match of a protein
must have
maxIniProbTH=0.99

# The minimum PeptideProphet score a peptide must have in order to be
even considered by Abacus
iniProbTH=0.50

# E.P.I: Experimental Peptide-probability Inclusion threshold
# If a protein does not contain at least one peptide exceeding this
PeptideProphet score, none of the
# peptide evidence for this protein will be considered. This is
applied on an experiment by experiment case.
epiTH=0

# The minimum ProteinProphet score a protein group must have in the
COMBINED file
minCombinedFilePw=0.90

# If true, Abacus will write ALL protein IDs belonging to a group in
the COMBINED file
# Protein IDs starting with ':::' are additional identifiers from the
same protein group in
```

```
# the COMBINED file. The representative protein for the group does not
start with ':::'
verboseResults=false

# The keep the HyperSQL database files that are created after the
program is done
keepDB=false

# Should the peptide weights be recalculated in the individual
experiment XML files.
# Useful for peptides that are highly degenerate within a single
protein groups
recalcPepWts=false

# Spectral count data will be reported in NSAF format.
# NSAF = _N_ormalized _S_pectral _A_bundance _F_actor
# For a detailed explanation of this method refer to this pubmed link:
# http://www.ncbi.nlm.nih.gov/pubmed/20166708
# Abacus reports NSAF values multiplied by a scaling factor. This is
done to
# control for numeric underflow (ie: really small numbers). The
scaling factor
# that is used is called the NSAF_FACTOR and is reported during
runtime in
# case you would like to rescale your data.
asNSAF=true

# If you are using decoy proteins in your searches, specify the first
few
# characters of the label indicating decoy proteins here
decoyTag=DECOY_

# Output format that will be produced by this parameter file
output=Default
```
